# Insights into How Calcium Forms Plaques in Arteries Pave the Way for New Treatments for Heart Disease

**DOI:** 10.1371/journal.pbio.1001533

**Published:** 2013-04-09

**Authors:** Janelle Weaver

**Affiliations:** Freelance Science Writer, Carbondale, Colorado, United States of America

Heart disease is the leading cause of death in the United States, and its primary cause is hardening of the arteries, or atherosclerosis. As people grow older, fat, cholesterol, and calcium build up in the walls of arteries and form hard structures called plaques. The process of calcium accumulation in blood vessels resembles bone formation and involves maintaining a balance between bone-forming cells called osteoblasts and bone-destroying cells called osteoclasts. The resulting plaques cause arteries to become narrow and stiff and can obstruct blood flow. As a consequence, oxygen-starved tissue can become damaged or die, leading to heart attack and stroke. Although many risk factors for atherosclerosis have been identified, the cause is not known and there is currently no way to reverse it once it sets in.

**Figure:**
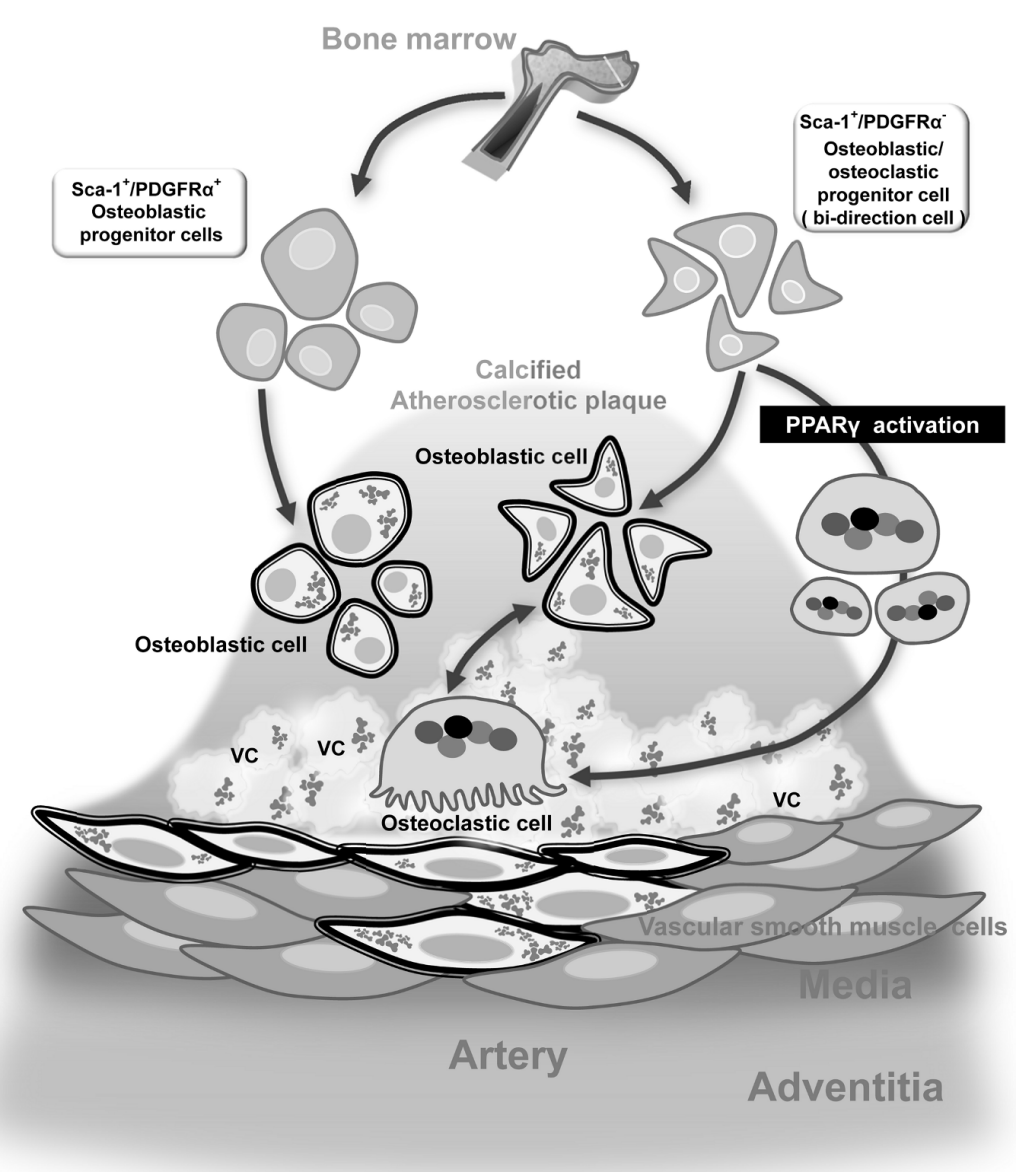
Illustration of calcifying progenitor cells and their proposed roles in atherosclerotic vascular calcification (VC). Sca-1+/PDGFRα- progenitor cells differentiate into osteoblasts/osteoclasts bi-directionally. PPARγ activation can shift the direction of Sca-1+/PDGFRα- progenitor cells toward osteoclasts.

In this issue of *PLOS Biology*, Hyo-Soo Kim of Seoul National University and his team characterize different types of cells that play a role in calcium accumulation in blood vessels. They report the novel concept that immature, stem-cell like cells have the potential to become either osteoblasts or osteoclasts, and that a drug can push these cells toward becoming osteoclasts instead of osteoblasts. The study offers new insights into how calcium builds up in the walls of blood vessels during advanced stages of atherosclerosis and paves the way for long-sought therapeutic strategies to combat this common problem.

To study the cause of calcium build-up in vessel walls, Kim and his team focused on calcifying progenitor cells—immature cells that can turn into specialized cells capable of either promoting or reversing calcium accumulation (osteoblasts or osteoclasts, respectively). They isolated these cells from the aortas of mice and sorted them into two groups. Both groups originated from bone marrow—spongy tissue found inside bones—and expressed a cell surface protein called stem cell antigen-1 (Sca-1), but only one group expressed another cell surface protein called platelet-derived growth factor receptor alpha (PDGFRα).

Moreover, both types of cells had a tendency to turn into osteoblast-like cells and thereby promote atherosclerotic calcium build-up. But cells expressing both Sca-1 and PDGFRα were more committed to the osteoblastic lineage, whereas those expressing only Sca-1 were bidirectional: they could also become osteoclast-like cells. The findings suggest that these bidirectional cells could be targeted by new therapies that shift their fate toward the osteoclastic lineage, thereby preventing calcium accumulation in blood vessels.

To test this idea, the researchers treated the bidirectional cells with a drug that stimulates a nuclear protein called peroxisome proliferator activated receptor-gamma (PPARγ), which is known to promote the formation of osteoclasts and inhibit the formation of osteoblasts. As expected, the treated cells primarily turned into osteoclast-like cells, suggesting that the drug could prevent and reverse calcium accumulation in blood vessels.

When the researchers injected bidirectional cells into a mouse model of atherosclerosis, they found an increase in the severity of calcium build-up and calcified plaques in arteries. But this effect was prevented by simultaneous treatment with the PPARγ-activating drug, which decreased the infiltration of osteoblasts into the plaques while increasing the infiltration of osteoclasts.

The study reveals the origin and features of calcifying cells found in blood vessels and establishes their roles in modulating calcium build-up in atherosclerosis, addressing an understudied topic and settling an active debate in the field. Moreover, the findings suggest that PPARγ activation can decrease atherosclerotic calcification by modulating the fate of bidirectional cells, opening up a new therapeutic avenue for reversing calcium accumulation in blood vessels.

**Cho H-J, Cho H-J, Lee H-J, Song M-K, Seo J-Y, et al. (2013) Vascular Calcifying Progenitor Cells Possess Bidirectional Differentiation Potentials. doi:10.1371/journal.pbio.1001534**

